# O Valor Preditivo do Índice Prognóstico Inflamatório para Detecção de No-Reflow em Pacientes com Infarto do Miocárdio com Supradesnivelamento do Segmento ST

**DOI:** 10.36660/abc.20230644

**Published:** 2024-04-15

**Authors:** Faysal Şaylık, Tufan Çınar, İbrahim Halil Tanboğa

**Affiliations:** 1 Health Sciences University Van Training and Research Hospital Department of Cardiology Van Turquia Health Sciences University, Van Training and Research Hospital, Department of Cardiology, Van – Turquia; 2 Health Sciences University Sultan II. Abdulhamid Han Training and Research Hospital Department of Cardiology Istanbul Turquia Health Sciences University, Sultan II. Abdulhamid Han Training and Research Hospital, Department of Cardiology, Istanbul – Turquia; 3 Hisar Intercontinental Hospital Department of Cardiology Istanbul Turquia Hisar Intercontinental Hospital, Department of Cardiology, Istanbul – Turquia; 4 Nisantasi University School of Health Science Department of Cardiology Istanbul Turquia School of Health Science, Nisantasi University, Department of Cardiology, Istanbul – Turquia; 5 Atatürk University Department of Biostatistics Erzurum Turquia Atatürk University, Department of Biostatistics, Erzurum – Turquia

**Keywords:** Fenômeno de não Refluxo, Infarto do Miocárdio com Elevação do Segmento ST, Intervenção Coronária Percutânea Primária, Nomograma

## Abstract

**Fundamento:**

O no-reflow (NR) é caracterizado por uma redução aguda no fluxo coronário que não é acompanhada por espasmo coronário, trombose ou dissecção. O índice prognóstico inflamatório (IPI) é um novo marcador que foi relatado como tendo um papel prognóstico em pacientes com câncer e é calculado pela razão neutrófilos/linfócitos (NLR) multiplicada pela razão proteína C reativa/albumina.

**Objetivo:**

Nosso objetivo foi investigar a relação entre IPI e NR em pacientes com infarto do miocárdio com supradesnivelamento do segmento ST (IAMCSST) submetidos a intervenção coronária percutânea primária (ICPp).

**Métodos:**

Um total de 1.541 pacientes foram incluídos neste estudo (178 com NR e 1.363 com refluxo). A regressão penalizada LASSO (Least Absolute Shrinkage and Select Operator) foi usada para seleção de variáveis. Foi criado um nomograma baseado no IPI para detecção do risco de desenvolvimento de NR. A validação interna com reamostragem Bootstrap foi utilizada para reprodutibilidade do modelo. Um valor de p bilateral <0,05 foi aceito como nível de significância para análises estatísticas.

**Resultados:**

O IPI foi maior em pacientes com NR do que em pacientes com refluxo. O IPI esteve associado de forma não linear com a NR. O IPI apresentou maior capacidade discriminativa do que o índice de imunoinflamação sistêmica, NLR e relação PCR/albumina. A adição do IPI ao modelo de regressão logística multivariável de base melhorou a discriminação e o efeito do benefício clínico líquido do modelo para detecção de pacientes com NR, e o IPI foi a variável mais proeminente no modelo completo. Foi criado um nomograma baseado no IPI para prever o risco de NR. A validação interna do nomograma Bootstrap mostrou uma boa capacidade de calibração e discriminação.

**Conclusão:**

Este é o primeiro estudo que mostra a associação de IPI com NR em pacientes com IAMCSST submetidos a ICPp.

**Figure:**
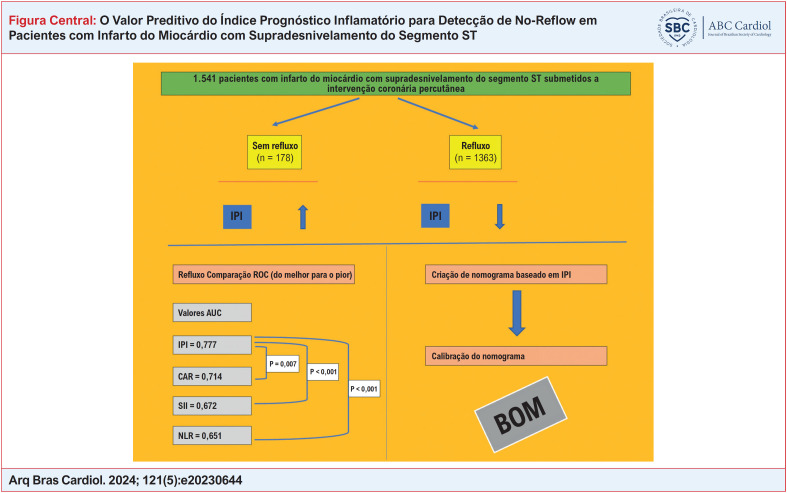


## Introdução

Atualmente, a modalidade de reperfusão recomendada em pacientes com infarto do miocárdio com supradesnivelamento do segmento ST (IAMCSST) é a intervenção coronária percutânea primária (ICPp) (IAMCSST).^1^ No entanto, apesar do fato de a ICPp restaurar com sucesso o fluxo coronário na artéria relacionada com o infarto (ARI) na maioria dos pacientes com IAMCSST, cerca de 5 a 15% desses pacientes não atingem um fluxo miocárdico e reperfusão adequados, o que é conhecido como fenômeno de não refluxo (NR).^2^ Na literatura atual, existem alguns estudos que demonstram os possíveis fatores de risco da NR, que incluem área isquêmica total, tempo isquêmico prolongado, hipertensão, tabagismo, dislipidemia, diabetes mellitus (DM) e aumento do estado inflamatório.^2^ A provável patogênese subjacente da NR inclui disfunção endotelial, bloqueio microvascular causado por espasmo microvascular distal e/ou microembolização e inflamação.^3^

A inflamação é reconhecida como a principal causa do fenômeno da NR, e vários marcadores inflamatórios têm sido propostos para a predição da NR. Índice de imunoinflamação sistêmica (SII),^4^ relação ácido úrico/albumina,^5^ relação proteína C reativa (PCR)/albumina (CAR)^6^ e relação neutrófilos/linfócitos^7^ são alguns dos preditores relatados na literatura. O índice prognóstico de inflamação (IPI) surgiu como um novo marcador inflamatório e é obtido por IPI=NLRxCAR . Um estudo recente demonstrou que a capacidade preditiva do IPI pode ser melhor do que a NLR e a CAR isoladamente. Como os níveis mais elevados de NLR e CAR estão associados ao desenvolvimento de NR, consideramos que a combinação de ambos os parâmetros, o IPI, pode detectar o NR com mais precisão do que qualquer parâmetro sozinho.^6 , 7^ Assim, nosso objetivo foi investigar a associação do IPI com o NR neste estudo.

## Material e métodos

Este estudo retrospectivo e transversal foi realizado em pacientes com IAMCSST que foram internados na clínica de cardiologia entre março de 2013 e março de 2022. O diagnóstico de IAMCSST foi feito com base em diretrizes recentes.^8^ Os critérios de exclusão foram os seguintes: receber terapia trombolítica, doença hepática ou renal grave, infecção ativa, doença autoimune crônica, doença hematológica, malignidades, uso de medicamentos que possam afetar os níveis de albumina e desnutrição. O estudo foi aprovado pelo comitê de ética local da nossa instituição e foi conduzido de acordo com a Declaração de Helsinque.

No momento da admissão, veias periféricas foram utilizadas para obtenção de amostras de sangue de cada paciente. Os parâmetros bioquímicos foram examinados por métodos convencionais, enquanto os parâmetros hematológicos foram avaliados por meio de um analisador hematológico (Beckman Coulter, FL, EUA). Antes da ICPp, as concentrações de albumina foram medidas pelo método Bromocresol Green. O IPI foi calculado com base na fórmula IPI=RNLxCAR .

### Angiografia coronária e ICPp

Utilizando uma abordagem radial ou femoral, um operador qualificado realizou angiografia coronária convencional (CAG). Antes da CAG, todos os pacientes receberam uma dose de ataque de inibidores P2Y12 e 300 mg de ácido acetilsalicílico. A mais recente diretriz IAMCSST da Sociedade Europeia de Cardiologia foi seguida durante as operações ICPp.^8^ Dois especialistas intervencionistas experientes, que desconheciam os dados dos pacientes, classificaram o fluxo TIMI na artéria relacionada ao infarto antes e depois da ICPp. Caso houvesse divergência entre eles, buscava-se a opinião do terceiro cardiologista e a decisão final era tomada com base na concordância de todos os cardiologistas. Para quantificar o fluxo TIMI após ICPp, foi utilizada a categorização do grau de fluxo trombólise no infarto do miocárdio (TIMI).^9^ Os fluxos TIMI 0, I e II na ausência de dissecção ou espasmo da artéria coronária foram definidos como fenômeno NR.^10^ O grau de perfusão miocárdica TIMI (TMPG) foi medido conforme descrito anteriormente.^11^ Um ou mais ramos da artéria relacionada ao infarto com nova deficiência de enchimento distal e bloqueio inesperado distal ao local da intervenção coronária foram identificados como embolização distal. Um NR eletrocardiográfico foi definido como a falta de resolução eletrocardiográfica do segmento ST> 70% no ECG.^12^ O tempo porta-balão foi definido como o tempo desde a admissão no pronto-socorro do centro de ICP até a insuflação do balão. Inibidores das glicoproteínas IIb/IIIa, adenosina e bloqueadores dos canais de cálcio, ou uma combinação desses medicamentos, foram utilizados no manejo da NR em nossa clínica. De acordo com o protocolo do hospital, a decisão de fazer trombectomia mecânica manual ficou a cargo do cardiologista responsável.

### Análise estatística

A normalidade das distribuições das variáveis foi verificada por meio do teste de Kolmogorov-Smirnov. Como todas as variáveis contínuas apresentaram distribuições não normais, a mediana (intervalo interquartil (IQR)) foi aplicada para apresentá-las. Números e porcentagens foram apresentados para variáveis categóricas. O teste do χ^2^ ou teste exato de Fisher foi calculado para as comparações das variáveis categóricas entre os grupos de estudo. As comparações das variáveis contínuas entre os grupos foram avaliadas pelo teste U de Mann-Whitney. A análise de regressão logística univariada foi utilizada para detectar variáveis estatisticamente significativas associadas ao NR (p<0,05). Para evitar *overfitting* e alcançar o desempenho ideal do modelo, a seleção de variáveis para análise de regressão logística multivariável foi feita com base na regressão penalizada LASSO. Um modelo multivariável com 10 variáveis selecionadas a partir da regressão LASSO foi construído para detectar preditores independentes de NR. Dois modelos foram criados como um modelo de linha de base (sem IPI) e um modelo completo (adicionando IPI ao modelo de linha de base). Os valores de probabilidade χ^2^ das variáveis no modelo multivariável foram utilizados para ordenar a proeminência das variáveis no modelo. A não linearidade foi verificada para todas as variáveis contínuas do modelo, e apenas o IPI foi associado de forma não linear ao desenvolvimento do IPI. Portanto, inserimos o IPI como um termo não linear usando um *spline* cúbico restrito no modelo multivariável. A análise da curva de características operacionais do receptor (ROC) foi utilizada para comparar as habilidades de discriminação do IPI com o SII e o modelo de linha de base com o modelo completo. Para comparar as curvas ROC foi utilizado o teste de De-long. Além disso, foram realizadas análises de curva de decisão para comparar os benefícios clínicos líquidos do IPI em relação ao SII e o modelo completo em relação ao modelo de base para obter um efeito aditivo do IPI. Foi construído um nomograma baseado no modelo completo para cálculo do risco previsto de NR. Foi utilizada uma validação interna usando 300 replicações de *bootstrap* , e as habilidades de discriminação e calibração do modelo foram avaliadas com estatística C, Dxy, pontuação de Brier, inclinação e parâmetros de interceptação. Além disso, um gráfico de calibração foi apresentado para mostrar a capacidade de predição dos nomogramas em novos dados clínicos. Programa R versão 3.6.3. (software estatístico R, Instituto de Estatística e Matemática, Viena, Áustria) foi utilizado para todas as análises estatísticas. O intervalo de confiança (IC) de 95% e um valor p bilateral de 0,05 foram utilizados para analisar os dados.

## Resultados

O resumo da metodologia e resultados do estudo está representado na Figura Central .

O estudo consistiu em 1.541 pacientes consecutivos com IAM (178 com NR e 1.363 com refluxo). A Tabela 1 representa as características demográficas, clínicas e laboratoriais basais de todos os pacientes. Pacientes com NR apresentaram maiores taxas de DM, estado de Killip ≥ 3, e valores mais elevados de contagem de leucócitos (leucócitos), plaquetas, neutrófilos, monócitos, largura de distribuição de glóbulos vermelhos, ácido úrico sérico, colesterol LDL, PCR, NLR, relação PCR/albumina, SII e IPI, e valores mais baixos da fração de ejeção do ventrículo esquerdo (FEVE), hemoglobina, linfócitos e albumina sérica quando comparados a pacientes com refluxo.

**Tabela 1:** Características demográficas, clínicas, laboratoriais e ecocardiográficas basais dos grupos de estudo

		Refluxo (N=1363)	No-reflow (N=178)	Valor p
Idade, anos		60,0 (52,0-68,0)	60,0 (47,0-73,5)	0,704
Sexo masculino, (%)		760 (55,8)	103 (57,9)	0,651
Índice de massa corporal, kg/m^2^		27,1 (24,5-30,1)	27,1 (24,1-29,8)	0,979
Diabetes mellitus, (%)		630 (46,2)	101 (56,7)	0,010
Hipertensão, (%)		680 (49,9)	96 (53,9)	0,350
Tabagismo, (%)		737 (54,1)	105 (59,0)	0,246
DAC anterior, (%)		615 (45,1)	80 (44,9)	1.000
DAC familiar, (%)		683 (50,1)	96 (53,9)	0,379
Killip > 3, (%)		291 (21,3)	63 (35,4)	<0,001
PA sistólica, mmHg		130 (120-130)	130 (120-130)	0,401
FEVE, %		45,0 (40,0-50,0)	40,0 (40,0-50,0)	<0,001
GB, × 109/L		9,96 (8,44-11,8)	11,5 (10,1-13,3)	<0,001
Hemoglobina, mg/dL		13,9 (12,9-14,9)	13,5 (12,5-14,6)	0,015
Plaquetas, × 109/L		226 (193-265)	230 (205-279)	0,024
Neutrófilos, × 109/L		7,36 (5,57-9,49)	8,73 (7,38-11,6)	<0,001
Linfócitos, × 109/L		1,90 (1,49-2,62)	1,65 (1,21-2,56)	0,002
Monócitos, × 109/L		0,51 (0,34-0,63)	0,57 (0,33-0,84)	<0,001
RDW, FL		44,9 (43,1-47,9)	46,2 (43,7-47,9)	<0,001
VPM, FL		10,2 (9,50-11,1)	10,3 (9,43-11,2)	0,851
Creatinina sérica, mg/dL		0,90 (0,80-1,00)	0,90 (0,80-1,00)	0,807
Ácido úrico sérico, mg/dL		5h00 (4h20-5h80)	5,50 (4,80-6,17)	<0,001
Sódio, mmol/L		139 (138-139)	138 (138-139)	0,498
Albumina, mg/dL		4h20 (4h00-4h30)	3,90 (3,50-4,00)	<0,001
Triglicerídeos, mg/dL		110 (88,5-155)	110 (95,8-143)	0,545
Colesterol HDL, mg/dL		44,0 (36,0-54,0)	41,0 (38,0-54,0)	0,410
Colesterol LDL, mg/dL		100 (78,0-130)	101 (80,0-140)	0,005
Colesterol total, mg/dL		183 (147-218)	192 (143-218)	0,172
PCR, mg/Dl		3,10 (2,00-5,10)	5,10 (3,60-6,77)	<0,001
NLR		3,74 (2,38-6,01)	5,50 (3,47-8,84)	<0,001
Relação PCR/albumina		0,78 (0,48-1,21)	1,32 (0,92-1,96)	<0,001
SII		815 (487-1438)	1310 (712-2216)	<0,001
IPI		2,93 (1,64-5,53)	7,19 (4,46-12,3)	<0,001
**Drogas**				
	AAS, (%)	622 (45,6)	84 (47,2)	0,755
	Antiagregantes, (%)	459 (33,7)	66 (37,1)	0,414
	Estatinas, (%)	600 (44,0)	83 (46,6)	0,563
	Inh. ECA/BRAs, (%)	552 (40,5)	80 (44,9)	0,292
	Bloqueadores beta, (%)	502 (36,8)	53 (29,8)	0,078
	Bloqueadores de canais de Ca, (%)	579 (42,5)	84 (47,2)	0,266

DAC: doença arterial coronariana; PA: pressão arterial; FEVE: fração de ejeção do ventrículo esquerdo; GB: glóbulos brancos; RDW: largura de distribuição de glóbulos vermelhos; VPM: volume plaquetário médio; HDL: lipoproteína de alta densidade; LDL: baixa densidade lipoproteína; PCR: proteína C reativa; NLR: relação neutrófilos-linfócitos; SII: índice imunológico inflamatório sistêmico; IPI: índice prognóstico inflamatório; AAS: ácido acetilsalicílico; ECA/BRA: enzima angiotensina/bloqueador de receptor de angiotensina.

**Figura 1:**
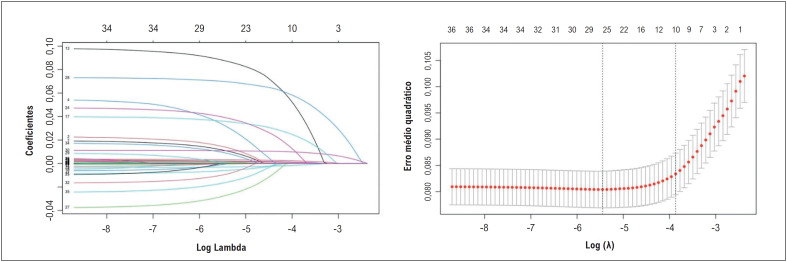
Regressão penalizada LASSO para seleção de variáveis.

**Figura 2:**
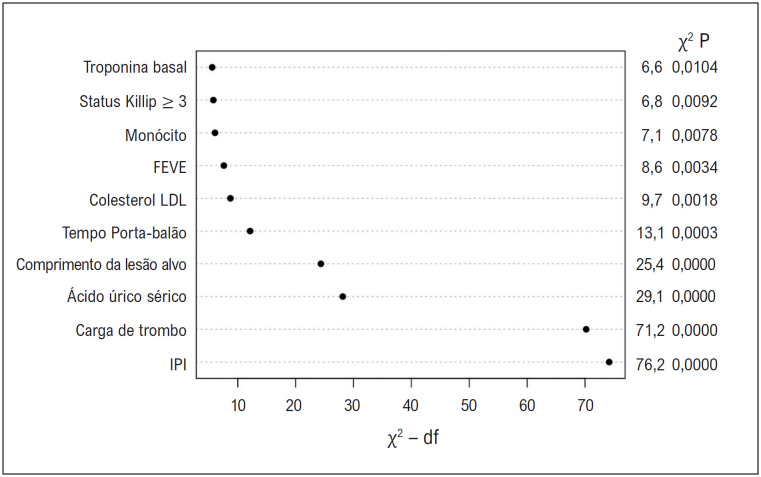
O tipo de variáveis com base nos valores de probabilidade χ^2^ para detectar a proeminência das variáveis no modelo multivariável. FEVE: fração de ejeção do ventrículo esquerdo; IPI: índice prognóstico inflamatório; LDL: lipoproteína de baixa densidade.

**Figura 3:**
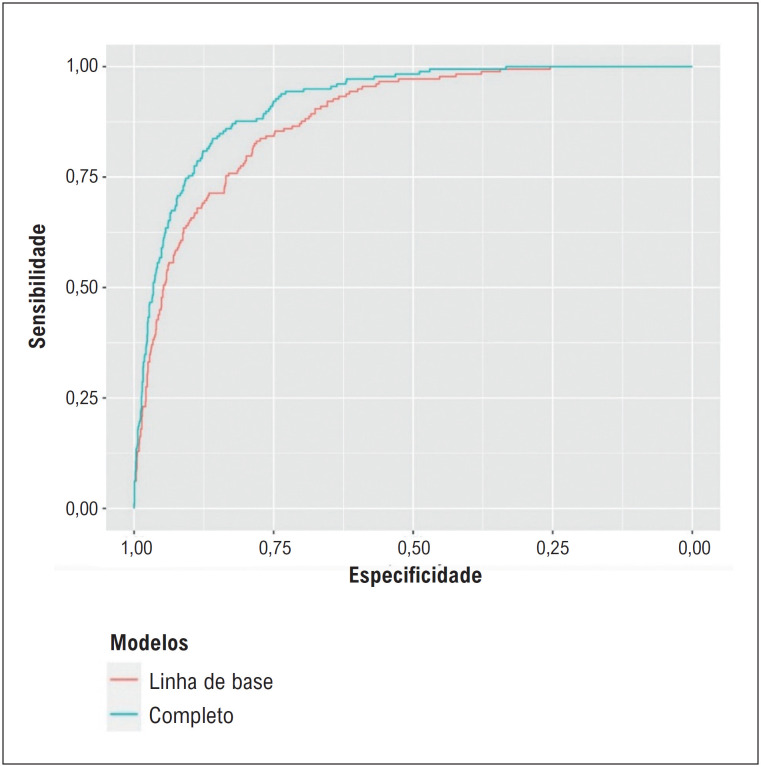
A comparação das habilidades discriminativas do modelo basal e completo usando curvas de características operacionais do receptor (ROC).

**Figura 4:**
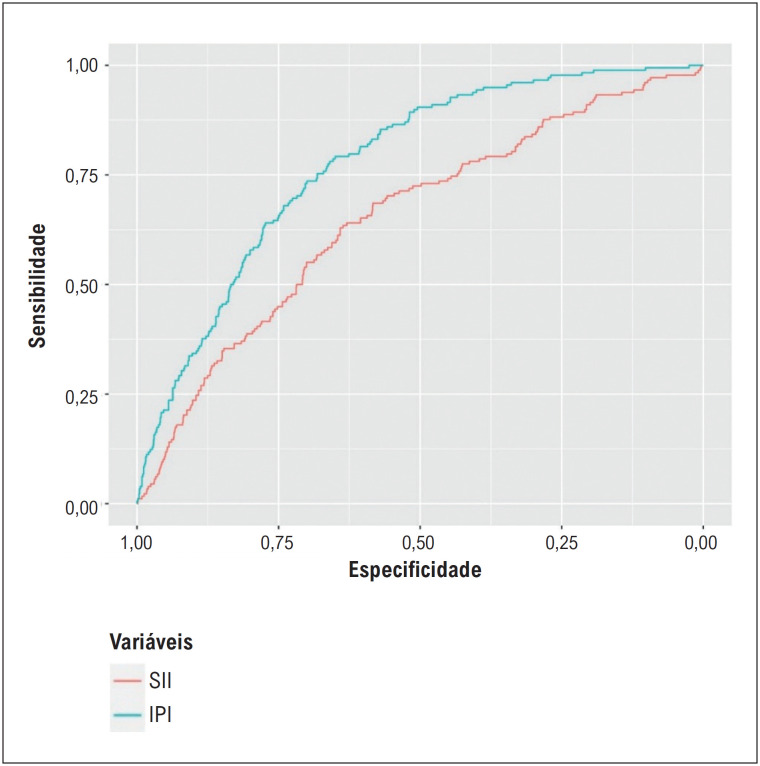
A comparação das habilidades discriminativas de IPI e SII usando curvas de características operacionais do receptor (ROC). IPI: índice prognóstico inflamatório.

**Figura 5:**
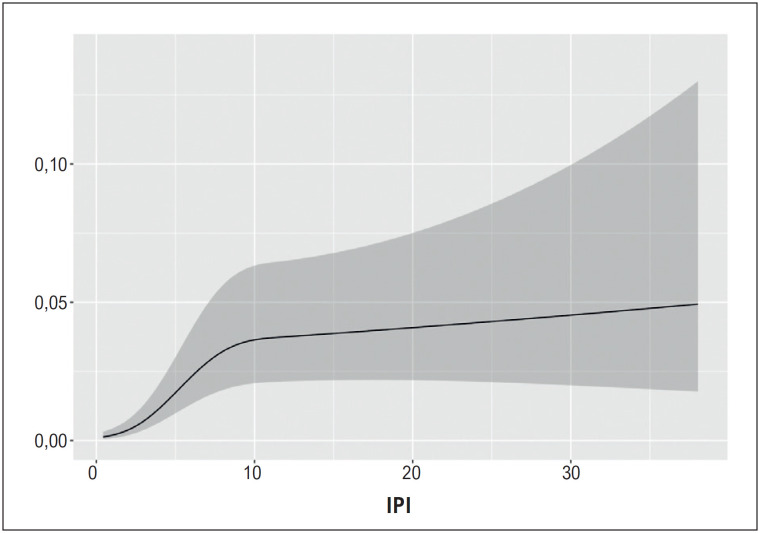
A relação não linear do IPI com o risco logarítmico de no-reflow. IPI: índice prognóstico inflamatório.

**Figura 6:**
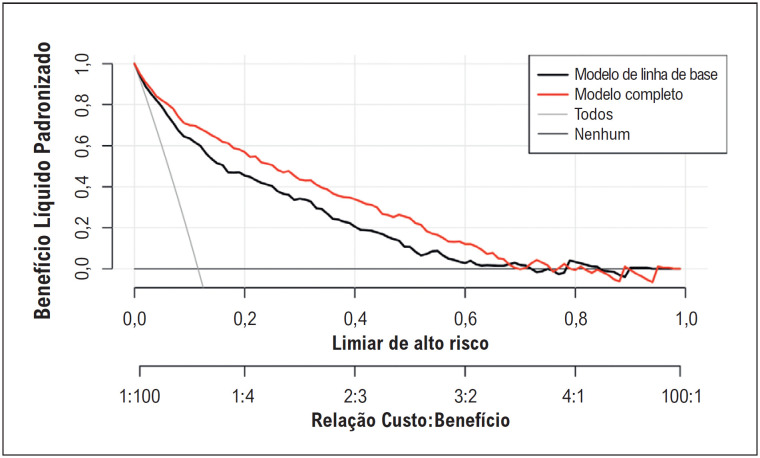
Análise da curva de decisão para detectar o benefício clínico líquido do IPI adicionando-o ao modelo de linha de base.

**Figura 7:**
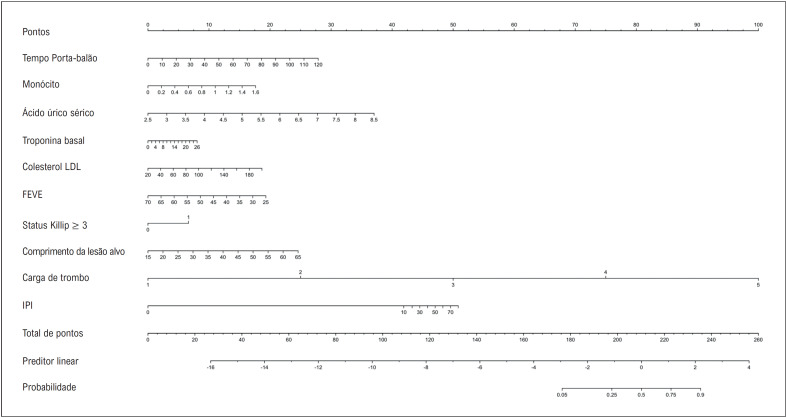
Nomograma clínico baseado no IPI para detectar o risco de desenvolvimento de no-reflow. FEVE: fração de ejeção do ventrículo esquerdo; IPI: índice prognóstico inflamatório.

**Figura 8:**
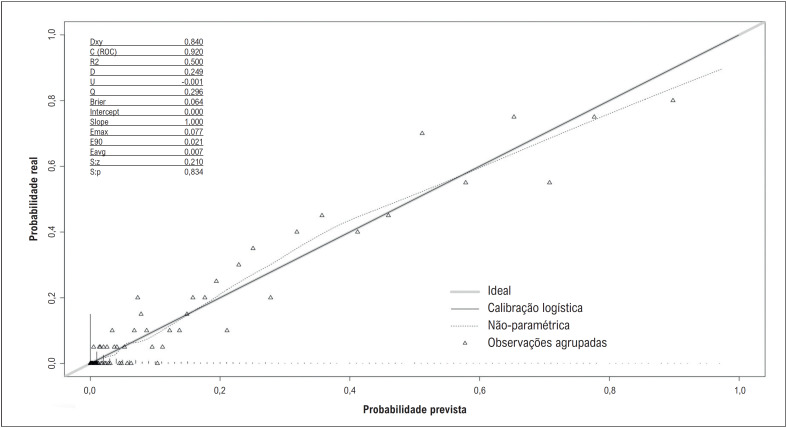
Gráfico de calibração do nomograma.

Tabela 2 mostra a comparação das características angiográficas dos grupos de estudo. O comprimento da lesão alvo e o tempo porta-balão foram maiores no grupo NR do que no grupo de refluxo. O grupo NR apresentou taxas mais altas de TMPG ≥2, embolização distal, grau de carga de trombo≥4, e taxas mais baixas de resolução ST do que o grupo de refluxo. O grupo NR apresentou maior mortalidade hospitalar do que o grupo de refluxo (14% vs. 4,1%, respectivamente, p = <0,001). Tempo porta-balão, contagem de monócitos, ácido úrico sérico, troponina I basal, colesterol LDL, FEVE, comprimento da lesão alvo, grau de carga de trombo, status de Killip e IPI, que foram selecionados pela regressão penalizada LASSO como proeminentes no modelo, foram utilizados no modelo multivariável ( Figura 1 ). Todas as variáveis do modelo foram independentemente associadas ao NR, e os resultados da análise de regressão logística multivariada foram apresentados como *odds ratio* para o intervalo interquartil (do percentil 25 ao 75) para variáveis contínuas ( Tabela 3 ). O modelo completo foi criado adicionando o IPI ao modelo de linha de base, e o IPI foi a variável mais proeminente no modelo (Probabilidade χ^2^ = 76,2, p <0,001) ( Figura 2 ). O modelo completo apresentou maior capacidade discriminativa do que o modelo basal para pacientes com NR de pacientes com refluxo (área sob a curva (AUC) = 0,919 vs. 0,883, respectivamente, valor p do teste De-long = 0,017) ( Figura 3 ). A capacidade discriminativa do IPI para pacientes com NR também foi superior ao SII (AUC = 0,777, 0,672, respectivamente, valor p do teste De-Long <0,001) ( Figura 4 ). Além disso, o IPI foi mais discriminativo do que ambos os componentes, incluindo NLR e CAR (valores de AUC = 0,777, 0,651, 0,714, respectivamente, valor p do teste De-Long para IPI vs. NLR <0,001, para IPI vs. CAR =0,007) (Arquivo complementar 1). Houve relação não linear entre o IPI e as chances de NR (p para não linearidade < 0,001) ( Figura 5 ). A análise da curva de decisão mostrou que a adição do IPI à linha de base melhorou o benefício clínico líquido acima de um valor limite de 2% ( Figura 6 ). O IPI apresentou maior benefício clínico líquido quando comparado ao SII acima do limite de 2% (arquivo complementar 2). Foi criado um nomograma clínico com variáveis do modelo multivariável para estratificação de risco de NR ( Figura 7 ). Um método de *bootstrappin* g gerando 300 amostras aleatórias a partir da distribuição amostral atual com reposição foi utilizado para a validação interna do nomograma, e os resultados mostraram uma boa calibração (R2 = 0,50, intercepto = 0, inclinação = 1, Emax = 0,08, Brier =0,06) e capacidade discriminativa (Dxy=0,84, estatística c=0,92) com otimismo ajustado. O gráfico de calibração também demonstrou a calibração adequada do nomograma ( Figura 8 ).

**Tabela 2:** Propriedades angiográficas e taxas de mortalidade hospitalar dos grupos de estudo

		Refluxo (N=1363)	No-reflow (N=178)	Valor p
**ARI**				0,085
	DAE, n (%)	708 (51,9)	93 (52,2)	
	Cx, n (%)	340 (24,9)	40 (22,5)	
	CD, n (%)	257 (18,9)	43 (24,2)	
	EVS, n (%)	58 (4,26)	2 (1,12)	
Diâmetro do vaso alvo ≥ 4 mm, n (%)		211 (15,5)	22 (12,4)	0,326
Comprimento da lesão alvo, mm		25,0 (20,0-38,0)	36,0 (25,0-40,0)	<0,001
Tempo porta-balão, min *		40,0 (30,0-45,0)	45,0 (30,0-60,0)	0,011
**Procedimento:**				0,080
	ICP + implante de stent, (%)	1207 (88,6)	166 (93,3)	
	Stent direto, (%)	114 (8,36)	11 (6,18)	
	Só ICP, (%)	42 (3,08)	1 (0,56)	
	TMPG ≥ 2, (%)	1078 (79,1)	110 (61,8)	<0,001
Embolização distal, n (%)		43 (3,15)	24 (13,5)	<0,001
Resolução do ST, n (%)		1311 (96,2)	138 (77,5)	<0,001
Grau de carga trombótica ≥ 4, n (%)		339 (24,9)	108 (60,7)	<0,001
Mortalidade hospitalar, n (%)		55 (4.1)	25 (14)	< 0,001

ARI: artéria relacionada ao infarto; DAE: artéria descendente anterior esquerda; Cx: circunflexa; CD: artéria coronária direita; EVS: enxerto venoso safeno; ICP: intervenção coronária percutânea; TMPG: trombólise no infarto do miocárdio (TIMI) grau de perfusão miocárdica.

* O tempo porta-balão foi definido como o tempo desde a admissão no pronto-socorro do centro de ICP até a insuflação do balão.

**Tabela 3:** Análise de regressão logística multivariável para detecção de não refluxo

Variáveis	OR	IC 95%	Valor p
Tempo porta-balão (30-46)	1.383	1.161-1.648	<0,001
Monócito (0,34-0,66)	1.360	1.084-1.706	0,007
Ácido úrico (4,2-5,8)	2.367	1.731-3.236	0,001
Troponina I basal (0,1-10)	1.307	1.065-1.603	0,011
Colesterol LDL (78-131)	1.615	1.195-2.183	0,018
FEVE (40-50)	0,688	0,535-0,883	0,003
Comprimento da lesão alvo (20-38)	2.165	1.603-924	<0,001
Grau de carga trombótica (4-5)	8.847	5.332-14.680	<0,001
Status Killip ≥ 3	1.784	1.154-759	<0,001
IPI (1,8-9,3)	10.564	5.989-18.632	<0,001

LDL: lipoproteína de baixa densidade; FEVE: fração de ejeção ventricular esquerda; IPI: índice prognóstico inflamatório; OR: razão de chances; IC: intervalo de confiança.

## Discussão

Este estudo mostrou que os pacientes com NR apresentavam um IPI mais elevado, e o IPI estava associado de forma não linear ao risco de desenvolvimento de NR. O IPI teve uma capacidade discriminativa mais elevada do que o SII, e a adição do IPI ao modelo de base melhorou a capacidade discriminativa do modelo e o efeito do benefício clínico líquido. Um nomograma de risco baseado no IPI apresentou boa capacidade discriminativa e preditiva na validação interna para detecção de NR. Por fim, o IPI foi a variável mais significativa no modelo multivariável.

A incidência do fenômeno NR pode variar de 3 a 15% em pacientes com IAMCSST, e a principal limitação da ICPp é o desenvolvimento do fenômeno NR na ARI.^1 , 3^ Em consonância com a literatura, a prevalência de NR na nossa amostra foi de 11,6%. Existem diversas complicações da NR, como arritmias e mortalidade.^1 , 3^ As taxas de mortalidade hospitalar dos grupos NR e de refluxo em nosso estudo foram de 14% vs. 4,1%, respectivamente, o que estava de acordo com relatos anteriores.^13 , 14^ Vários fatores de risco foram identificados para o desenvolvimento de NR após ICPp, incluindo atraso no tempo de ICPp, menor FEVE, maior comprimento da lesão-alvo, maiores graus de trombos e pior estado de Killip.^2 , 15 , 16^ Descobrimos que maior tempo porta-balão, baixa FEVE, maior comprimento da lesão-alvo, maior grau de trombo e pior estado de Killip foram preditores independentes de NR e todos foram utilizados no nomograma. Muitas pesquisas foram realizadas para determinar possíveis fatores de risco para NR; no entanto, ainda falta um método fiável de avaliação dos riscos.^3 , 17^ Portanto, nosso objetivo foi desenvolver um nomograma de predição de risco baseado no IPI no presente estudo. Com base em nosso conhecimento, esta é a primeira pesquisa a avaliar a associação de IPI com NR em pacientes com IAM na literatura.

Os mecanismos subjacentes responsáveis da NR não foram totalmente compreendidos. No entanto, a oclusão microvascular devido ao acúmulo de plaquetas e neutrófilos, a compressão externa que ocorre após edema miocárdico e a vasoconstrição grave poderiam ser esperadas como as principais causas.^3^ A inflamação desempenha um papel fundamental no desenvolvimento da NR. Sabe-se que o tônus microvascular, o tônus epicárdico e a função dos neutrófilos são afetados pela inflamação crônica de baixo grau. A estimulação e o acúmulo de neutrófilos polimorfonucleares surgem no miocárdio lesionado logo após a reperfusão da ARI.^18^ A deformabilidade celular pode ser ainda mais reduzida durante a ativação de neutrófilos. Essas características hemorreológicas poderiam ser um fator no aprisionamento de leucócitos nos capilares, o que resultaria em obstrução microvascular.^18^

A relação entre marcadores inflamatórios e NR foi investigada anteriormente. Wang et al. mostraram que a contagem de neutrófilos na admissão foi um preditor independente de NR.^19^ Dogan et al. observaram que contagens baixas de linfócitos estavam relacionadas à NR.^20^ Wagdy et al. combinaram esses dois fatores hematológicos e relataram que a RNL foi maior na NR e foi um preditor independente de NR.^7^ Outro marcador inflamatório, a PCR, foi relatado como maior em pacientes com NR, e a PCR foi independentemente associada à NR.^15^ A PCR pode aumentar o risco de NR de duas maneiras possíveis: em primeiro lugar, níveis elevados de PCR estimulam a hipercoagulação, o que resulta em oclusão microvascular e, em segundo lugar, leva a um grande tamanho do infarto, aumentando a cascata do complemento.^21 - 23^

A albumina é um reagente negativo de fase aguda e possui efeitos anti-inflamatórios e antioxidantes.^24^ Um estado inflamatório mais elevado está relacionado com níveis mais baixos de albumina sérica.^25^ A diminuição da albumina pode induzir lesão de reperfusão miocárdica. O desenvolvimento de um estado de hipercoagulabilidade na luz capilar pode ser influenciado pela perda das propriedades antioxidantes da albumina na microcirculação coronariana.^26^ Finalmente, descobriu-se que a albumina mais baixa está associada à aterosclerose coronariana prolongada.^27^ Kurtul et al. relataram que a albumina sérica mais baixa estava associada à NR e ao GPM mais baixo em pacientes com IAM após ICPp.^28^ A PCR e a albumina foram combinadas e descobriu-se que o CAR estava independentemente ligado à NR.^6^

O IPI emergiu como um novo marcador inflamatório que é um composto de NLR e CAR. Foi relatado como um preditor de prognóstico em pacientes com câncer.^29 , 30^ Nenhum estudo avaliou o IPI no IAMCSST para NR na literatura. Espera-se que a combinação de variáveis tenha maior capacidade preditiva do que os parâmetros separadamente. Esta pesquisa indicou que o IPI tinha maior capacidade discriminativa e preditiva do que NLR e CAR. Além disso, o SII é um dos marcadores inflamatórios mais relatados na literatura. Também encontramos a superioridade do IPI sobre o SII na detecção de NR nesta pesquisa.

O IPI, um marcador facilmente calculável a partir do hemograma periférico, e também o nomograma baseado no IPI podem prever o desenvolvimento do fenômeno NR e podem ser usados para estratificação de risco e ajudar os médicos a tomar decisões para o tratamento de pacientes com IAM submetidos a ICPp que estão em alta risco para o desenvolvimento de NR. Em pacientes com alto risco de NR com base no IPI antes da ICPp, os médicos devem estar cientes da realização de procedimentos que levem a um menor risco de desenvolvimento de NR, incluindo implantação direta de stent sem usar dilatações repetidas de balão, usar balões revestidos com medicamento, usar glicoproteínas Inibidores IIb/IIIa, transferências rápidas de pacientes para os centros com unidades de ICPp para obter tempos curtos de dor até o balão, usando dispositivos de aspiração de trombos, bem como usando stent longo único em vez de stents sobrepostos.

Houve algumas limitações deste estudo, em primeiro lugar, devido ao desenho do estudo transversal e retrospectivo, uma relação causal entre o IPI e o NR não pôde ser bem documentada. Em segundo lugar, pode haver efeitos de confusão não medidos, apesar da presença de um modelo de regressão multivariável. Em terceiro lugar, outra desvantagem foi a ausência de técnicas mais precisas para determinar o grau de NR, como a ressonância magnética coronariana e a ecocardiografia com contraste miocárdico. Em quarto lugar, os resultados não puderam ser generalizados para outros pacientes com síndrome coronariana aguda porque apenas pacientes com IAMCSST foram incluídos no estudo. Em quinto lugar, como a duração do estudo foi longa e foram observadas algumas mudanças nas estratégias de tratamento para o manejo de pacientes com IAMCSST, tais diferenças não foram levadas em consideração em nosso estudo. Portanto, novos estudos que investiguem o valor preditivo do IPI em diferentes anos poderão esclarecer esta questão.

## Conclusão

Este estudo revelou que o IPI foi um preditor independente de NR em pacientes com IAMCSST. O IPI pode ser um marcador melhor que o SII, NLR e CAR para detectar pacientes com NR. Finalmente, o nomograma baseado no IPI apresentou boas propriedades de discriminação e calibração para estratificação de risco.

## Supplementary Material



## *Material suplementar

Para informação adicional do Material Suplementar, por favor, clique aqui .
