# Supplementary figures and images for: O Valor Preditivo do Índice Prognóstico Inflamatório para Detecção de No-Reflow em Pacientes com Infarto do Miocárdio com Supradesnivelamento do Segmento ST
Source: Arq Bras Cardiol. 2024 Apr 15;121(4):e20230644. [Article in Portuguese] doi: 10.36660/abc.20230644 (PMC11081176; doi:10.36660/abc.20230644)

Supplementary 1

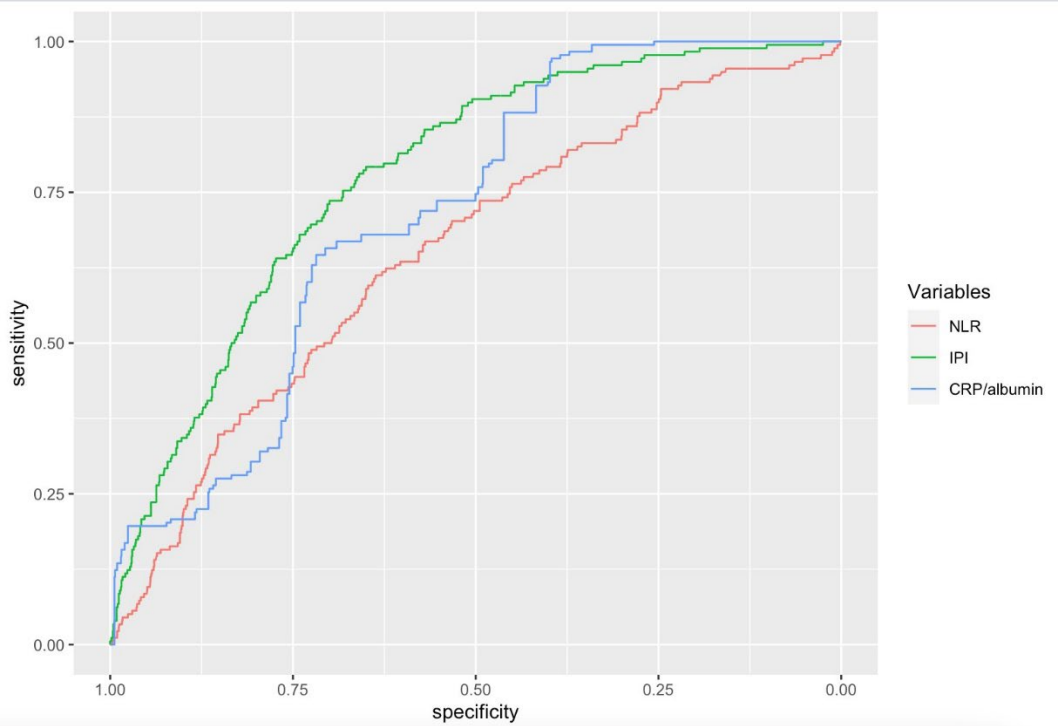

Supplementary 2

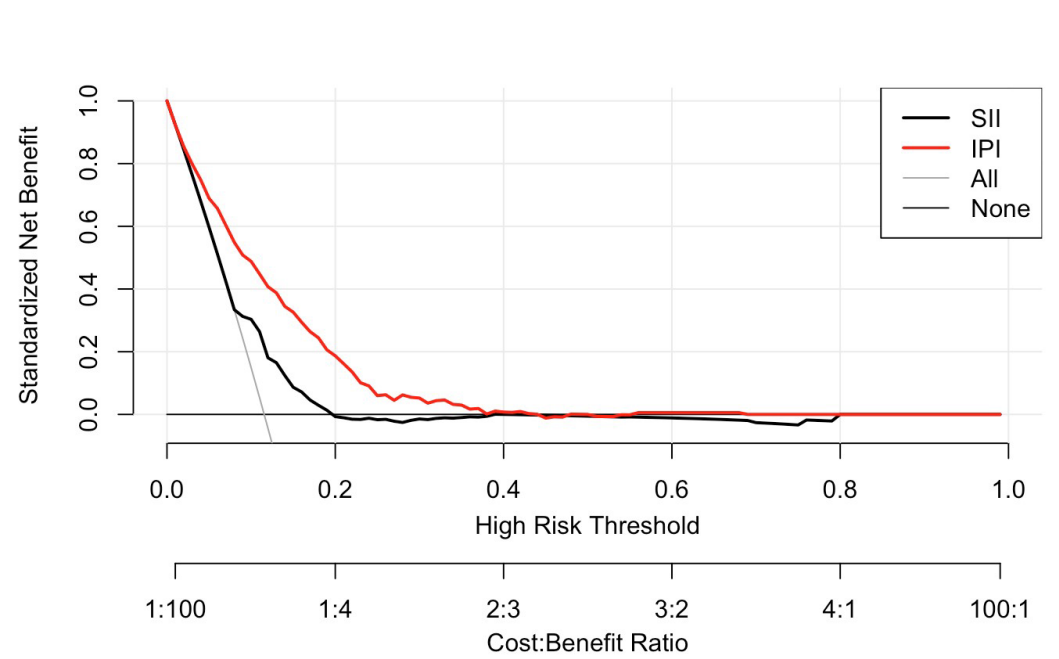

Supplement: Supplementary file 1 [file 0066-782X-abc-121-04-e20230644-Suppl01.pdf]
