# Supplementary material for: O Valor Preditivo do Índice Prognóstico Inflamatório para Detecção de No-Reflow em Pacientes com Infarto do Miocárdio com Supradesnivelamento do Segmento ST
Source: Arq Bras Cardiol. 2024 Apr 15;121(4):e20230644. [Article in Portuguese] doi: 10.36660/abc.20230644 (PMC11081176; doi:10.36660/abc.20230644)
Supplement: Translation

# Translation: The Predictive Value of the Inflammatory Prognostic Index for Detecting No-Reflow in ST-Elevation Myocardial Infarction Patients

**DOI:** 10.36660/abc.20230644i

**Authors:** Faysal Şaylık, Tufan Çınar, İbrahim Halil Tanboğa

**Affiliations:** 1 Van Turkey Health Sciences University, Van Training and Research Hospital, Department of Cardiology, Van – Turkey; 2 Istanbul Turkey Health Sciences University, Sultan II. Abdulhamid Han Training and Research Hospital, Department of Cardiology, Istanbul – Turkey; 3 Istanbul Turkey Hisar Intercontinental Hospital, Department of Cardiology, Istanbul – Turkey; 4 Istanbul Turkey School of Health Science, Nisantasi University, Department of Cardiology, Istanbul – Turkey; 5 Erzurum Turkey Atatürk University, Department of Biostatistics, Erzurum – Turkey

**Keywords:** No-Reflow Phenomenon, ST Elevation Myocardial Infarction, Percutaneous Coronary Intervention, Nomograms

## Abstract

**Background:**

No-reflow (NR) is characterized by an acute reduction in coronary flow that is not accompanied by coronary spasm, thrombosis, or dissection. Inflammatory prognostic index (IPI) is a novel marker that was reported to have a prognostic role in cancer patients and is calculated by neutrophil/lymphocyte ratio (NLR) multiplied by C-reactive protein/albumin ratio.

**Objective:**

We aimed to investigate the relationship between IPI and NR in ST-segment elevation myocardial infarction (STEMI) patients undergoing primary percutaneous coronary intervention (pPCI).

**Methods:**

A total of 1541 patients were enrolled in this study (178 with NR and 1363 with reflow). Lasso panelized shrinkage was used for variable selection. A nomogram was created based on IPI for detecting the risk of NR development. Internal validation with Bootstrap resampling was used for model reproducibility. A two-sided p-value <0.05 was accepted as a significance level for statistical analyses.

**Results:**

IPI was higher in patients with NR than in patients with reflow. IPI was non-linearly associated with NR. IPI had a higher discriminative ability than the systemic immune-inflammation index, NLR, and CRP/albumin ratio. Adding IPI to the baseline multivariable logistic regression model improved the discrimination and net-clinical benefit effect of the model for detecting NR patients, and IPI was the most prominent variable in the full model. A nomogram was created based on IPI to predict the risk of NR. Bootstrap internal validation of nomogram showed a good calibration and discrimination ability.

**Conclusion:**

This is the first study that shows the association of IPI with NR in STEMI patients who undergo pPCI.

## Introduction

Currently, the recommended reperfusion modality in patients with ST-elevation myocardial infarction (STEMI) is primary percutaneous coronary intervention (pPCI) (STEMI).^1^However, despite the fact that pPCI successfully restores coronary flow in the infarct-related artery (IRA) in the majority of STEMI patients, roughly 5 to as high as 15% of such patients do not achieve an adequate myocardial flow and reperfusion, which is referred to as the no-reflow (NR) phenomenon.^2^In the current literature, there are some studies demonstrating the possible risk factors of NR, which include total ischemic area, prolonged ischemic time, hypertension, smoking, dyslipidemia, diabetes mellitus (DM), and enhanced inflammatory status.^2^The probable underlying pathogenesis of NR includes endothelial dysfunction, microvascular blockage driven by distal microvascular spasm and/or microembolization, and inflammation.^3^

The inflammation is recognized as the main cause of NR phenomenon, and several inflammatory markers have been proposed for the prediction of NR. Systemic immune-inflammation index (SII),^4^uric acid/albumin ratio,^5^C-reactive protein (CRP)/albumin ratio (CAR),^6^and neutrophil/lymphocyte ratio^7^are some of the reported predictors in the literature. Inflammation prognostic index (IPI) has emerged as a new inflammatory marker and is gained by IPI=NLRxCAR . A recent study has demonstrated that the predictive capability of IPI might be better than NLR and CAR alone. Because the higher levels of NLR and CAR are associated with NR development, we consider that the combination of both parameters, the IPI, might detect the NR more accurately than either parameter alone.^6 , 7^Thus, we aimed to investigate the association of IPI with NR in this study.

## Material and Methods

This retrospective, cross-sectional study was conducted on STEMI patients who were admitted to the cardiology clinic between March 2013 and March 2022. STEMI diagnosis was made based on recent guidelines.^8^The exclusion criteria were as follows: receiving thrombolytic therapy, severe hepatic or renal disease, active infection, chronic autoimmune disease, hematological disease, malignancies, taking drugs that might affect albumin levels, and malnutrition. The study was approved by the local ethics committee of our institution and was conducted according to the Helsinki Declaration.

At the time of admission, peripheral veins were used to obtain blood samples for each patient. The biochemical parameters were examined using conventional methods, whereas the hematologic parameters were assessed using a hematology analyzer (Beckman Coulter, town, FL, USA). Prior to pPCI, albumin concentrations were measured using the Bromocresol Green method. IPI was calculated based on the formula IPI=NLRxCAR .

### Coronary angiography and pPCI

Using either a radial or femoral approach, a qualified operator performed conventional coronary angiography (CAG). Prior to CAG, all patients received a loading dose of P2Y12 inhibitors and 300 mg of acetylsalicylic acid. The most recent European Society of Cardiology STEMI guideline was followed during the pPCI operations.^8^Two experienced interventional specialists, who were unaware of the patients’ data, graded the TIMI flow in the infract-related artery before and after pPCI. If there was a disagreement between them, the opinion of the third cardiologist was sought, and the final decision was made based on the agreement of all cardiologists. To quantify the TIMI flow after pPCI, the thrombolysis in myocardial infarction (TIMI) flow grade categorization was used.^9^TIMI flows 0, I, and II in the absence of coronary artery dissection or spasm were defined as NR phenomenon.^10^TIMI myocardial perfusion grade (TMPG) was measured as described previously.^11^One or more branches of the infarct-related artery with a new distal filling deficiency and an unexpected blockage distal to the coronary intervention site were identified as distal embolization. An electrocardiographic NR was defined as the lack of >70% electrocardiographic ST-segment resolution in the ECG.^12^Door-to-balloon time was defined as the time from admission to the emergency department of the PCI center to balloon inflation. Glycoproteins IIb/IIIa inhibitors, adenosine, and calcium channel blockers, or a combination of these drugs were used in the management of NR in our clinic. According to hospital protocol, the decision to do a manual mechanical thrombectomy was left to the attending cardiologist.

### Statistical analysis

The normality of the distributions of variables was checked using the Kolmogorov-Smirnov test. Because all continuous variables had non-normal distributions, the median (interquartile range (IQR)) was applied to present them. Numbers and percentages were presented for categorical variables. χ^2^test or Fisher's exact test was calculated for the comparisons of categorical variables between study groups. Comparisons of continuous variables between the groups were assessed using the Mann-Whitney U test. Univariable logistic regression analysis was used to detect statistically significant variables associated with NR (p<0.05). To avoid overfitting and achieve optimal model performance, variable selection for multivariable logistic regression analysis was done based on Lasso penalized shrinkage regression. A multivariable model with 10 variables selected from Lasso regression was built to detect independent predictors of NR. Two models were created as a baseline model (without IPI) and a full model (by adding IPI to the baseline model). Likelihood χ^2^values of variables in the multivariable model were used to sort the prominence of variables in the model. Non-linearity was checked for all continuous variables in the model, and only IPI was non-linearly associated with the development of IPI. Therefore, we input IPI as a non-linear term using a restricted cubic spline in the multivariable model. Receiver operating characteristics (ROC) curve analysis was used to compare the discrimination abilities of IPI with SII and the baseline model with the full model. To compare the ROC curves, the De-long test was employed. Also, decision curve analyses were performed to compare the net clinical benefits of IPI over SII and the full model over the baseline model to gain an additive effect of IPI. A nomogram was built based on the full model for the calculation of the predicted risk of NR. An internal validation using 300 bootstrap replications was used, and the discrimination and calibration abilities of the model were evaluated with C-statistic, Dxy, Brier score, slope, and intercept parameters. Also, a calibration plot was presented to show the prediction capability of nomograms in new clinical data. R-program version 3.6.3. (R statistical software, Institute for Statistics and Mathematics, Vienna, Austria) was utilized for all statistical analyses. The 95 % confidence interval (CI) and a 2-sided p-value of 0.05 were used to analyze the data.

## Results

The summary of the methodology and results of the study is represented in the Central Illustration .

The study consisted of 1541 consecutive STMI patients (178 with NR and 1363 with reflow). Table 1 represents the baseline demographic, clinical, and laboratory characteristics of all patients. Patients with NR had higher rates of DM, Killip status ≥ 3, and higher values of white blood cell count (WBC), platelets, neutrophils, monocytes, red cell distribution width, serum uric acid, LDL-cholesterol, CRP, NLR, CRP/albumin ratio, SII, and IPI, and lower values of left ventricular ejection fraction (LVEF), hemoglobin, lymphocytes, and serum albumin when compared to patients with reflow.

**Table 1:** Baseline demographic, clinical, laboratory, and echocardiographic features of study groups

		Reflow (N=1363)	No-reflow (N=178)	p-value
Age, years		60.0 (52.0-68.0)	60.0 (47.0-73.5)	0.704
Male gender, (%)		760 (55.8)	103 (57.9)	0.651
Body mass index, kg/m^2^		27.1 (24.5-30.1)	27.1 (24.1-29.8)	0.979
Diabetes mellitus, (%)		630 (46.2)	101 (56.7)	0.010
Hypertension, (%)		680 (49.9)	96 (53.9)	0.350
Cigarette smoking, (%)		737 (54.1)	105 (59.0)	0.246
Previous CAD, (%)		615 (45.1)	80 (44.9)	1.000
Familial CAD, (%)		683 (50.1)	96 (53.9)	0.379
Killip > 3, (%)		291 (21.3)	63 (35.4)	<0.001
Systolic BP, mmHg		130 (120-130)	130 (120-130)	0.401
LVEF, %		45.0 (40.0-50.0)	40.0 (40.0-50.0)	<0.001
WBC, × 109/L		9.96 (8.44-11.8)	11.5 (10.1-13.3)	<0.001
Hemoglobin, mg/dL		13.9 (12.9-14.9)	13.5 (12.5-14.6)	0.015
Platelets, × 109/L		226 (193-265)	230 (205-279)	0.024
Neutrophils, × 109/L		7.36 (5.57-9.49)	8.73 (7.38-11.6)	<0.001
Lymphocytes, × 109/L		1.90 (1.49-2.62)	1.65 (1.21-2.56)	0.002
Monocytes, × 109/L		0.51 (0.34-0.63)	0.57 (0.33-0.84)	<0.001
RDW, fL		44.9 (43.1-47.9)	46.2 (43.7-47.9)	<0.001
MPV, fL		10.2 (9.50-11.1)	10.3 (9.43-11.2)	0.851
Serum creatinine, mg/dL		0.90 (0.80-1.00)	0.90 (0.80-1.00)	0.807
Serum uric acid, mg/dL		5h00 (4h20-5h80)	5.50 (4.80-6.17)	<0.001
Sodium, mmol/L		139 (138-139)	138 (138-139)	0.498
Albumin, mg/dL		4h20 (4h00-4h30)	3.90 (3.50-4.00)	<0.001
Triglycerides, mg/dL		110 (88.5-155)	110 (95.8-143)	0.545
HDL cholesterol, mg/dL		44.0 (36.0-54.0)	41.0 (38.0-54.0)	0.410
LDL cholesterol, mg/dL		100 (78.0-130)	101 (80.0-140)	0.005
Total cholesterol, mg/dL		183 (147-218)	192 (143-218)	0.172
CRP, mg/Dl		3.10 (2.00-5.10)	5.10 (3.60-6.77)	<0.001
NLR		3.74 (2.38-6.01)	5.50 (3.47-8.84)	<0.001
CRP/albumin ratio		0.78 (0.48-1.21)	1.32 (0.92-1.96)	<0.001
SII		815 (487-1438)	1310 (712-2216)	<0.001
IPI		2.93 (1.64-5.53)	7.19 (4.46-12.3)	<0.001
**Drugs**				
	ASA, (%)	622 (45.6)	84 (47.2)	0.755
	Anti-aggregants, (%)	459 (33.7)	66 (37.1)	0.414
	Statins, (%)	600 (44.0)	83 (46.6)	0.563
	ACE inh/ARBs, (%)	552 (40.5)	80 (44.9)	0.292
	Beta-blockers, (%)	502 (36.8)	53 (29.8)	0.078
	Ca channel blockers, (%)	579 (42.5)	84 (47.2)	0.266

CAD: coronary artery disease; BP: blood pressure; LVEF: left ventricle ejection fraction; WBC: white blood cell; RDW: red cell distribution width; MPV: mean platelet volume; HDL: high-density lipoprotein; LDL: low-density lipoprotein; CRP: C-reactive protein; NLR: neutrophil-lymphocyte ratio; SII: systemic inflammatory immune index; IPI: inflammatory prognostic index; ASA: acetylsalicylic acid; ACE/ARB: angiotensin enzyme/angiotensin receptor blocker.

Table 2 demonstrates the comparison of angiographic features of the study groups. Target lesion length and door-to-balloon time were longer in the NR group than in the reflow group. The NR group had higher rates of TMPG ≥ 2, distal embolization, thrombus burden grade ≥ 4, and lower rates of ST resolution than the reflow group. The NR group had higher in-hospital mortality than the reflow group (14 % vs. 4.1 %, respectively, p = <0.001). Door-to-balloon time, monocyte count, serum uric acid, baseline troponin I, LDL-cholesterol, LVEF, Target lesion length, thrombus burden grade, Killip status, and IPI, which were selected by Lasso penalized shrinkage regression as prominent in the model, were used in the multivariable model ( Figure 1 ). All variables in the model were independently associated with NR, and the results from multivariable logistic regression analysis were displayed as the odds ratio for the interquartile range (from the 25^th^to the 75^th^percentile) for continuous variables ( Table 3 ). The full model was created by adding IPI to the baseline model, and IPI was the most prominent variable in the model (Likelihood χ^2^= 76.2, p <0.001) ( Figure 2 ). The full model had higher discriminative ability than the baseline model for patients with NR from patients with reflow (area under curve (AUC)=0.919 vs. 0.883, respectively, De-long test p-value =0.017) ( Figure 3 ). The discriminative capability of IPI for patients with NR was also higher than SII (AUC = 0.777, 0.672, respectively, De-Long test p-value <0.001) ( Figure 4 ). Furthermore, IPI was more discriminative than both the components, including NLR and CAR (AUC values = 0.777, 0.651, 0.714, respectively, De-Long test p-value for IPI vs. NLR <0.001, for IPI vs. CAR =0.007) (Supplementary file 1). There was a non-linear relationship between IPI and the odds of NR (p for non-linearity < 0.001) ( Figure 5 ). Decision curve analysis showed that adding IPI to the baseline improved the net clinical benefit above a threshold value of 2% ( Figure 6 ). IPI had higher net clinical benefit when compared to SII above a threshold of 2% (Supplementary file 2). A clinical nomogram with variables in the multivariable model was created for the risk stratification of NR ( Figure 7 ). A bootstrapping method by generating 300 random samples from the current sample distribution with replacement was used for the internal validation of the nomogram, and the results showed a good calibration (R2=0.50, intercept= 0, slope=1, Emax=0.08, Brier=0.06) and discriminative ability (Dxy=0.84, c-statistic=0.92) with adjusted optimism. The calibration plot also demonstrated the proper calibration of the nomogram ( Figure 8 ).

**Table 2:** Angiographic properties and in-hospital mortality rates of study groups

		Reflow (N=1363)	No-reflow (N=178)	p-value
IRA				0.085
	LAD, n (%)	708 (51.9)	93 (52.2)	
	Cx, n (%)	340 (24.9)	40 (22.5)	
	RCA, n (%)	257 (18.9)	43 (24.2)	
	SVG, n (%)	58 (4.26)	2 (1.12)	
Target vessel diameter ≥ 4mm, n (%)		211 (15.5)	22 (12.4)	0.326
Target lesion length, mm		25.0 (20.0-38.0)	36.0 (25.0-40.0)	<0.001
Door to balloon time, min *		40.0 (30.0-45.0)	45.0 (30.0-60.0)	0.011
**Procedure**				0.080
	PCI + stenting, (%)	1207 (88.6)	166 (93.3)	
	Direct stenting, (%)	114 (8.36)	11 (6.18)	
	Only PCI, (%)	42 (3.08)	1 (0.56)	
	TMPG ≥ 2, (%)	1078 (79.1)	110 (61.8)	<0.001
Distal embolization, n (%)		43 (3.15)	24 (13.5)	<0.001
ST-resolution, n (%)		1311 (96.2)	138 (77.5)	<0.001
Thrombus burden grade ≥ 4, n (%)		339 (24.9)	108 (60.7)	<0.001
In-hospital mortality, n (%)		55 (4.1)	25 (14)	< 0.001

IRA: infract-related artery; LAD: left anterior descending artery; Cx: circumflex; RCA: right coronary artery; SVG: saphenous venous graft; PCI: percutaneous coronary intervention; TMPG: thrombolysis in myocardial infarction (TIMI) myocardial perfusion grade.

* Door-to-balloon time was defined as the time from admission to the emergency department of the PCI center to balloon inflation.

**Figure 1:** Lasso penalized shrinkage for variable selection.

**Table 3:** Multivariable logistic regression analysis for detecting no-reflow

Variables	OR	95% CI	p-value
Door to balloon time (30-46)	1.383	1.161-1.648	<0.001
Monocyte (0.34-0.66)	1.360	1.084-1.706	0.007
Uric acid (4.2-5.8)	2.367	1.731-3.236	0.001
Baseline troponin I (0.1-10)	1.307	1.065-1.603	0.011
LDL cholesterol (78-131)	1.615	1.195-2.183	0.018
LVEF (40-50)	0.688	0.535-0.883	0.003
Target lesion length (20-38)	2.165	1.603-924	<0.001
Thrombus burden grade (4-5)	8.847	5.332-14.680	<0.001
Killip status ≥ 3	1.784	1.154-759	<0.001
IPI (1.8-9.3)	10.564	5.989-18.632	<0.001

OR: odds ratio; CI: confidence interval; LDL: low-density lipoprotein; LVEF: low ventricle ejection fraction; IPI: inflammatory prognostic index.

**Figure 2:** The sort of variables based on likelihood χ^2^values to detect the prominence of variables in the multivariable model. LDL: low-density lipoprotein; LVEF: low ventricle ejection fraction; IPI: inflammatory prognostic index.

**Figure 3:** The comparison of discriminative abilities of baseline and full model using receiver operating characteristics (ROC) curves.

**Figure 4:** The comparison of discriminative abilities of IPI and SII using receiver operating characteristics (ROC) curves. IPI: inflammatory prognostic index.

**Figure 5:** The non-linear relationship of IPI with log-odds risk of no-reflow. IPI: inflammatory prognostic index.

**Figure 6:** Decision curve analysis to detect net clinical benefit of IPI by adding to baseline model.

**Figure 7:** Clinical nomogram based on IPI for detecting the risk of no-reflow development. LDL: low-density lipoprotein; LVEF: low ventricle ejection fraction; IPI: inflammatory prognostic index.

**Figure 8:** Calibration plot of nomogram.

## Discussion

This study showed that NR patients had a higher IPI, and the IPI was non-linearly associated with the risk of NR development. IPI had a higher discriminative ability than SII, and adding IPI to the baseline model improved the model's discriminative capacity and net clinical benefit effect. A risk nomogram based on IPI had good discriminative and predictive ability in internal validation for detecting NR. Finally, IPI was the most significant variable in the multivariable model.

The incidence of the NR phenomenon might range from 3 to 15% in STEMI patients, and the main limitation of pPCI is the development of the NR phenomenon in the IRA.^1 , 3^In line with the literature, the prevalence of NR in our sample was 11.6%. There are several complications of NR, such as arrhythmias and mortality.^1 , 3^In-hospital mortality rates of NR and reflow groups in our study were 14% vs. 4.1%, respectively, which were in accordance with previous reports.^13 , 14^Several risk factors were identified for the development of NR after pPCI, including delayed pPCI time, lower LVEF, longer target lesion length, higher thrombus grades, and worse Killip status.^2 , 15 , 16^We found that longer door-to-balloon time, low LVEF, longer target lesion length, higher thrombus grade, and worse Killip status were independent predictor of NR and all were used in the nomogram. Lots of research has been carried out to determine possible risk factors for NR; however, a reliable risk assessment method is still lacking.^3 , 17^Therefore, we aimed to develop a risk prediction nomogram based on IPI in the current study. Based on our knowledge, this is the first research to evaluate the association of IPI with NR in STMI patients in the literature.

The responsible underlying mechanisms of NR have not been fully understood. Nonetheless, microvascular occlusion due to platelet and neutrophil accumulation, external compression occurring after myocardial edema and severe vasoconstriction could be expected as being the main causes.^3^Inflammation plays a key role in the development of NR. Microvascular tone, epicardial tone, and neutrophil function are all known to be impacted by chronic low-grade inflammation. Polymorphonuclear neutrophil stimulation and accumulation arise in the injured myocardium soon after IRA reperfusion.^18^Cellular deformability can be further reduced during neutrophil activation. These hemorrheologic characteristics could be a factor in leukocyte trapping in capillaries, which would result in micro-vascular plugging.^18^

The relationship between inflammatory markers and NR has been investigated previously. Wang et al. showed that neutrophil count on admission was an independent predictor of NR.^19^Dogan et al. noted that low lymphocyte counts were related to NR.^20^Wagdy et al. combined those two hematologic factors and reported that NLR was higher in NR and was an independent predictor of NR.^7^Another inflammatory marker, CRP, was reported to be higher in NR patients, and CRP was independently associated with NR.^15^CRP might enhance the NR risk in two possible ways: firstly, high CRP levels encourage hypercoagulation which results in microvascular occlusion, and secondly, it leads a large infarct size by driving up complement cascade.^21 - 23^

Albumin is a negative acute phase reactant and has anti-inflammatory and antioxidant effects.^24^Higher inflammatory status is related to lower serum albumin levels.^25^Albumin decrease might induce myocardial reperfusion injury. The development of a hypercoagulable status in the capillary lumen may be influenced by the loss of antioxidant properties of albumin in the coronary microcirculation.^26^Finally, lower albumin was found to be associated with extended coronary atherosclerosis.^27^Kurtul et al. reported that lower serum albumin was associated with NR and lower MPG in STMI patients after pPCI.^28^CRP and albumin were combined, and CAR was found to be independently linked with NR.^6^

The IPI has emerged as a new inflammatory marker that is a composite of both NLR and CAR. It has been reported as a prognostic predictor in cancer patients.^29 , 30^No study has evaluated the IPI in STEMI for NR in the literature. The combination of variables is expected to have a higher predictive ability than the parameters separately. This research indicated that IPI had higher discriminative and predictive ability than both NLR and CAR. Furthermore, SII is one of the most reported inflammatory markers in the literature. We also found the superiority of IPI over SII in detecting NR in this research.

The IPI, an easily calculable marker from peripheral blood count, and also IPI-based nomogram might predict the development of NR phenomenon and could be used for risk stratification and help clinicians to make decisions for the management of STMI patients undergoing pPCI who are at high risk for NR development. In patients with a high risk for NR based on the IPI before pPCI, clinicians should be aware of performing procedures that lead to lower risk of the development of NR including direct stent implantation without using repeated balloon dilatations, using drug-coated balloons, using glycoproteins IIb/IIIa inhibitors, quick transfers of patients to the centers with pPCI units for gaining short pain-to-balloon times, using thrombus aspiration devices as well as using single long stent instead of overlap stents.

There were some limitations of this study as follows. Firstly, due to the cross-sectional and retrospective study design, a causal relationship between the IPI and NR could not be well documented. Secondly, there might be unmeasured confounding effects despite the presence of a multivariable regression model. Thirdly, another drawback was the absence of more accurate techniques to determine the degree of NR, such as coronary magnetic resonance imaging and myocardial contrast echocardiography. Fourthly, the results could not be generalized to other patients with acute coronary syndrome because only STEMI patients were included in the study. Fifthly, because the study duration was long and some changes in the treatment strategies for the management of STEMI patients were observed, such differences were not taken into account in our study. Therefore, further studies investigating the predictive value of IPI at different years might shed light on this issue.

## Conclusion

This study revealed that IPI was an independent predictor of NR in STEMI patients. IPI might be a better marker than SII, NLR, and CAR for detecting NR patients. Finally, IPI based nomogram had good discrimination and calibration properties for risk stratification.

## *Supplemental Materials

For additional information Supplemental Material, please click here .
